# Pathogen-host interaction mediated by vesicle-based secretion in schistosomes

**DOI:** 10.1007/s00709-020-01515-y

**Published:** 2020-05-27

**Authors:** Miriam Bischofsberger, Franziska Winkelmann, Anne Rabes, Emil C. Reisinger, Martina Sombetzki

**Affiliations:** Department of Tropical Medicine, Infectious Diseases and Section of Nephrology, University Medical Center Rostock, Rostock, Germany

**Keywords:** *Schistosoma mansoni*, Extracellular vesicles, EV based communication, Parasite host interaction

## Abstract

As part of the parasite’s excretory/secretory system, extracellular vesicles (EVs) represent a potent communication tool of schistosomes with their human host to strike the balance between their own survival in a hostile immunological environment and a minimal damage to the host tissue. Their cargo consists of functional proteins, lipids, and nucleic acids that facilitate biological processes like migration, nutrient acquisition, or reproduction. The most important impact of the vesicle-mediated communication, however, is the promotion of the parasite survival via mimicking host protein function and directly or indirectly modulating the immune response of the host. Overcoming this shield of immunological adaption in the schistosome-host relation is the aim of current research activities in this field and crucial for the development of a reliable anti-schistosomal therapy. Not least because of their prospective use in clinical applications, research on EVs is now a rapidly expanding field. We herein focus on the current state of knowledge of vesicle-based communication of schistosomes and discussing the role of EVs in facilitating biological processes and immune modulatory properties of EVs considering the different life stages of the parasite.

## Introduction

Billions of people worldwide are hosts of parasites, which contribute to a large extent to the global health burden of infectious origin (Torgerson et al. 2015). It is believed that hundreds of helminth species and protozoa can affect humans. Only a small portion of these pathogens are responsible for the world’s most important and debilitating parasitic diseases, e.g., malaria, Chagas’ disease, and schistosomiasis (Montaner et al. 2014). Critical for all internal parasites, regardless of the kingdom they belong to, is their obligatory host dependence for survival and reproduction. They often undergo multiple life cycle transformations with asexual and sexual replication phases in several hosts (Mantel and Marti 2014). This survival mode requires not only an enormous ability to adapt to different environmental conditions but also a high resistance to the various defense mechanisms of the hosts. To hijack their host organisms and make them useful for their specific needs, worms have developed into master manipulators of the immune system during co-evolutionary development with their specific hosts. For this purpose, parasites use extracellular vesicles (EV) as an effective tool for inter-cellular, inter-tissue, and cross-organism communication. Formally considered as garbage-bins, EVs are today recognized as extended arm of the parasite for balancing their own survival and the host pathology. The cargo of EVs consists of functional proteins, lipids, nucleic acids used to regulate tissue repair, neural communication, transfer of pathogenic proteins, and regulation of immune responses (Marcilla et al. 2012; Zhang et al. 2013). EVs appear as exosomes, microvesicles, apoptotic bodies, and other cell-derived membrane-enclosed vesicles (Fig. 2). Exosomes are synthesized through reverse budding of the late endosomal membrane. This results in the formation of multivesicular bodies (MVB) (Gustafson et al. 2017; Rodriguez-Boulan et al. 2005). Via fusion of MVBs with the outer membrane, exosomes are released into the extracellular environment (Piper and Katzmann 2007; Li et al. 2015). Subsequently they exert their biological function via (i) binding of target cells mediated by specific ligand-receptor recognition and triggering downstream signaling, or (ii) by directly delivering cargo due to rapid fusion with the target cell membrane (van Dongen et al. 2016). This is a very basic description of the numerous types of vesicles. Not least due to the absence of appropriate isolation techniques of these heterogeneous membrane-bound carriers, questions regarding the molecular mechanisms behind intercellular communication, tagging and merging with target cells, specific delivering of cargo, etc. cannot be answered in detail so far. It is obvious that the communication between parasite and host is not only unidirectional. Almost every cell can produce and release EV under healthy (Raposo and Stoorvogel 2013) and pathological conditions (Hristov et al. 2004). In the context of a parasitic infection, host-derived EVs efficiently activate immune responses. It has been shown that plasma cell-derived vesicles induce CD40 on antigen-presenting cells in response to an infection with *Plasmodium berghei* and therefore driving potential T cell priming and effector initiation for subsequent parasite eradication (Couper et al. 2010). In other studies, it has been shown that *Leishmania major* or *Toxoplasma gondii* pulsed dendritic cells form exosomes that induce protective Th1 immunity against these parasites (Aline et al. 2004; Coakley et al. 2016).

**Fig. 1:**
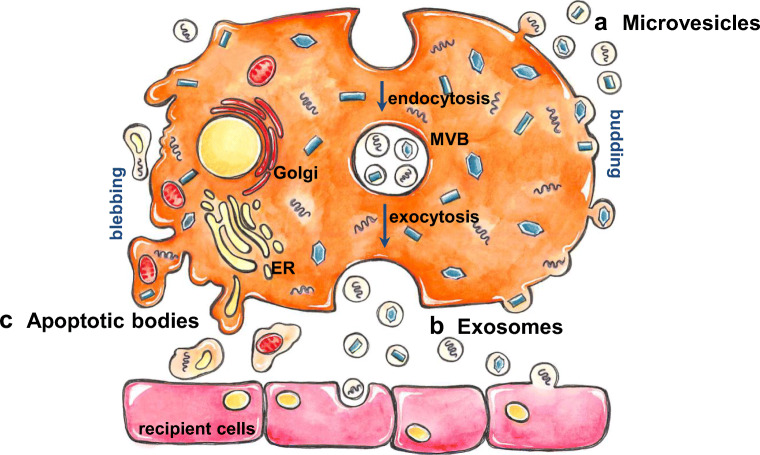
Biogenesis of extracellular vesicles. Broadly categorized, there are three types of vesicles: (**A**) Microvesicles are originated by outward budding and fusion of the plasma membrane; (**B**) exosomes arise from fusion of multivesicular bodies (MVB) with the plasma membrane. MVB derive from developing early to late endosomes. Inward budding of endosomal membrane forms intraluminal vesicle that are released upon fusion with the plasma membrane, (**C**) apoptotic blebs are released by cells undergoing apoptosis (modified from Gustafson et al. 2017)

Schistosomiasis, caused by blood flukes (trematode worms) of the genus *Schistosoma* spp., is considered the most important helminthic disease in terms of morbidity and mortality, with more than 240 million infected people worldwide (Andrade 2009). The parasites invade humans and other mammalian hosts via skin and migrate through the lungs to the mesenteric blood vessels where they reach maturity as adult worms (Gryseels et al. 2006). Disease symptoms include spleno- and hepatomegaly, periportal fibrosis, portal hypertension (*S. mansoni*), urinary obstruction, bladder carcinoma (*S. haematobium*), and sterility. By virtue of serious health and social consequences for the chronically affected people and the growing concerns over resistance, there is continued pressure to identify and validate new schistosome drug targets and vaccine candidates (Fonseca et al. 2015). Characteristic of infection with schistosomes, and helminths in general, are the longevity of the parasites within the mammalian host, repeated re-infections due to less or no induction of immunity, and selective immune suppression to prevent protective Th2 responses (Taylor et al. 2012). As other helminths, schistosomes employ EVs to enable migration through the body, maturation, and immune evasion. Every life-stage of schistosomes has developed its own ingenious skills to communicate with the host via EVs for the maintenance of biological functions, and the creation of a “parasite-friendly” environment (Fig. 1).

## Cercariae to skin stage

**Fig. 2:**
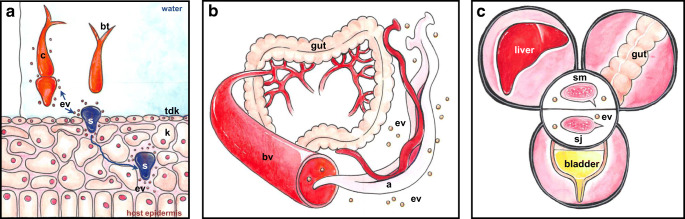
Life stages of schistosomes affecting the host via vesicle-based communication. (**A**) Aquatic cercaria (c) is attracted to the human skin (tdk, terminally differentiated keratinocytes) by chemotrophic host signaling. During skin invasion the bifurcated tail (bt) is detached to initiate formation of the schistosomulum (s). Extracellular vesicles (ev) contain molecules, which support penetration of the membrane, and migration of the newly transformed schistosomulum (modified from Egesa et al. 2018). (**B**) Intravascular adult worms (a) secrete extracellular vesicles to coordinate migration within blood vessels (bv), nutrient acquisition, maintenance of hemostasis, and egg production. (**C**) miRNA containing extracellular vesicles (ev) from parasite eggs (sm, *S. mansoni*; sj, *S. japonicum*) can transfer their cargo to recipient cells. Apart of facilitating life stage-specific functions, all schistosome life stages use the vesicular signaling to modulate the host immune system in favor of their own survival

Aquatic cercariae represent the infectious life stage of the parasite. The main challenge of cercariae consists in the invasion of the mammalian host skin and dealing with innate immune defense mechanisms of the host. They are considered as complement sensitive in contrast to later larvae stages (Braschi et al. 2006). Cercariae are 500 μm long and consist of an anterior region (oral sucker), the body segment and the typical bifurcated tail. *S. mansoni* cercariae have two pairs of preacetabular and three pairs of postacetabular glands. Since there is no de novo synthesis of proteins by cercariae, gland contents have to be pre-formed in earlier parasite life stages (Harrop and Wilson 1993). Right after the attachment to the skin, cercariae start transformation into schistosomula or “skin-stage.” Therefore, it is difficult to classify the source of the vesicles secreted (Fig. 2).

Acetabular glands are a source of several serine proteases with elastase activity, which among others, are capable to degrade macromolecules in the skin. Proteases of *S. mansoni* cercariae are either packed in EVs or are secreted as soluble ES products. They form small tissue gaps within the skin and surround the cercariae like a protective wall or “smoke screen” (Mountford and Trottein 2004; Jenkins et al. 2005; Curwen et al. 2006). The most abundant proteases found in glandular secretions of *S. mansoni* are cercarial elastase (SmCE). However, *S. japonicum* cercariae are distinguishable from cercariae of other *Schistosoma* species in regard to the success (lower host specificity) and speed of migration through the skin (Ruppel et al. 2004). It is assumed that in regard to *S. japonicum*, other classes of proteases are involved in host invasion (Ingram et al. 2012). SmPepM8, a metalloprotease of the leishmanolysin family, was found to be another abundant constituent of *S. mansoni* cercarial secretions. In leishmaniosis leishmanolysin enhances skin passage of the parasite. A similar function for the *S. mansoni* version of this protein can be expected. The same applies to another secreted cercarial protease, SmDPP IV. SmDPP IV is known to be involved in skin invasion processes in other organisms, which implies a similar function in *S. mansoni* infection (Curwen et al. 2006; Silva et al. 2012). A chymotrypsin-like serine protease (SmCE), secreted by glands, is capable to cleave a variety of human skin macromolecules, including insoluble elastin (Salter et al. 2000). The function of a 28-kDa glutathione-S-transferase (Sm28GST) is associated with detoxification processes of parasite and host derived products and is involved in controlling parasite fertility. Together with Sm28GST, *S. bovis* 28-GST and other molecules, like Sb14–3-3, are currently being intensively studied as potential vaccine candidates (Hansell et al. 2008; Riveau et al. 2018; de la Torre-Escudero et al. 2016).

Most of the excretory/secretory (E/S) products of transforming *S. mansoni* cercariae are proteins with immunomodulatory potential (Jenkins et al. 2005). Fluorescence labelling of E/S products (proteases or esterases) of transforming *S. mansoni* cercariae resulted in clearly visible vesicles released by cercarial acetabular glands. Macrophages and dendritic cells are able to incorporate the labeled E/S material via an active actin and Ca2+-dependent phagocytic process. In vitro and in vivo, this mechanism has shown to trigger the development of Th2 polarizing dendritic cells (Paveley et al. 2009). Glycomic analysis of E/S products of transforming cercariae and eggs revealed the Lewis X (Lex) as one major terminal structure. Lex is a strong inducer of immune modulators like interkeukin-10 and prostaglandin (E2) (Jang-Lee et al. 2007). Lex-containing glycoconjugates drive maturation of native dendritic cells (DC) to a DC type 2 phenotype in a toll-like receptor 4–dependent manner, consequently leading to Th2-type responses (Curwen et al. 2006; Silva et al. 2012). Proteomic analysis of *S. mansoni* acetabular gland vesicles identified different cercarial elastase species, as well as paramyosin and SPO-1 (Sm16) (Knudsen et al. 2005). Paramyosin is a well-known inhibitor of the complement membrane attacking complex (MAC) and the classical complement activation pathway by C1q binding (Deng et al. 2003). Paramyosin binding of complement factors C8 and C9 leads to blockage of MAC formation and finally cell lysis. Anti-inflammatory SPO-1 or SmSPO-1, known to be expressed in sporocysts (an earlier snail stage of schistosomes), is a major protein secreted via EVs in cercariae and schistosomula (Rao and Ramaswamy 2000). In vitro, SPO-1 downregulates interleukin 1α expression in human keratinocytes, prevents lymphoproliferation, and suppresses intercellular adhesion molecule-1 (ICAM-1) expression in endothelial cells (Ramaswamy et al. 1997). Following invasion of the skin, cercariae shed their immunogenic outer membrane, the glycocalyx, which initiates transformation of cercariae into the next larvae stage, i.e., the schistosomulum. This process is mediated by a 26-kDa serine protease found in secreted exosome-like vesicles. Here it is important to mention that cercarial EVs itself activate MAC to lyse vesicles and release vesicle contents to the environment (Da’dara and Krautz-Peterson 2014). MAC initiates the rupture of vesicles along the cercarial surface, facilitating release of vesicle contents and thus enables shedding of the glycocalyx and migration through the skin layers. This clearly demonstrates of how cercariae use a part of the host’s innate immune defense system to their own advantage.

## Schistosomula

The most abundant molecules found in E/S products of schistosomula are involved in stress response, carbohydrate metabolism, and protein degradation (Cao et al. 2016a, b). This reflects the challenges they have to face within the human body which are the following: adaption from an aerobic to an anaerobic metabolism, defending host immune responses, migration through the skin, and changing of their body structure.

Schistosomal EVs are originated from unicellular acetabular or head glands (Nowacki et al. 2015b). Proteome analysis of exosome-like vesicle contents of *S. mansoni* schistosomula revealed tetraspanins, heat shock proteins (HSP), annexins, Rab 11 proteins, 14-3-3 protein isoforms, cytoskeletal proteins, and metabolic enzymes. For not all of the proteins found in vesicles a function has been defined yet. Due to their abundance in other organisms, however, functions can often be deducted (Table 1). Glycerinaldehyde-3-phosphate (GAPDH), present in *S. mansoni* schistosomal EVs (Nowacki et al. 2015b), binds to plasminogen and thus facilitates the invasion and migration of schistosomes.

**Table 1:** Assumed functions of proteins found in different Schistosoma species and life stages

Protein	Species	Described function	Ref.
Cercariae			
Paramyosin	*S. mansoni*	Immunogenic, inhibitor of the complement system	(Knudsen et al. 2005)
SPO-1 (Sm16)	*S. mansoni*	Anti-inflammatory, downregulates IL1-α in keratinocytes, prevents lymphoproliferation, suppresses ICAM-1 on endothelial cells	(Knudsen et al. 2005)
FBA	*S. mansoni*	Granuloma downregulation	(Marques et al. 2008)
Myosin heavy chain	*S. mansoni*	Potent stimulator of IFN-γ of CD4^+^ cells	(Eberl et al. 2000)
PEPCK	*S. mansoni*	Induce a balanced Th1/Th2 response	(Rutitzky et al. 2001)
GAPDH	*S. japonicum, S. mansoni*	Inhibitor of the complement system (binding of plasminogen and complement factor C3)	(Cao et al. 2016a, 2016b)
Calcium ATPase	*S. mansoni*	Control of calcium homeostasis	(Noël et al. 2001)
Tropomyosin	*S. mansoni*	Cytoskeletal protein	(MacGregor and Shore 1990)
TPI	*S. japonicum,* *S. mansoni*	Converts glyceraldehyde-3-phosphate to dehydroxyacetone phosphate, a key reaction in glycolysis	(Sun et al. 1999) (dos Reis et al. 1993)
GST	*S. mansoni*	Associated with detoxification processes of parasite, involved in controlling parasite fertility	(Hansell et al. 2008)
CE	*S. mansoni*	Cleaves skin macromolecules e.g. insoluble elastin	(Salter et al. 2000; Ingram et al. 2011)
Calpain	*S. mansoni*	Cytoskeletal remodeling	(Fox et al. 1990)
Schistosomula			
Rab11	*S. mansoni,* *S. japonicum*	Fusion of multi-vesicular bodies (MVB) with the plasma membrane	(Beckett et al. 2013)
CD63 antigen	*S. mansoni,* *S. japonicum*	Formation/cargo sorting of intraluminal vesicles in MVBs	(Baietti et al. 2012)
Syntaxin	*S. mansoni*	Q-SNARE, mediate vesicle-fusion	(Koles et al. 2012)
Calpain	*S. mansoni*	Cytoskeletal remodeling	(Fox et al. 1990)
Adult worms			
TSP-2	*S. japonicum*	Member of tetraspanins, linked to the immune evasion of schistosomes and tegument turnover, structural organization of the tegument	(Tran et al. 2010; Cai et al. 2008)
VAMP2	*S. japonicum*	Member of SNAREs, linked to membrane fusion, maintenance of tegument, glucose uptake, worm development and egg production	(Han et al. 2017)
VIP36	*S. mansoni*	L-type lectin, might participate in the complex secretory activity within the egg envelope and tegument protein	(Ornelas et al. 2017)
Calpain	*S. mansoni*	extracellular calpain activity, cleaving fibronectin	(Wang et al. 2017)
ATPDase-1	*S. mansoni*	Hydrolyzes extracellular prothrombotic ATP and ADP, inhibiting platelet aggregation and activation	(Da’Dara and Skelly 2014)
Enolase	*S. mansoni*	Binds plasminogen and promote its activation	(Figueiredo et al. 2015a, 2015b)
Sm22.6	*S. mansoni*	Inhibition of human thrombin	(Lin and He 2006)
SP2	*S. mansoni*	Trypsin-like protease, activates plasminogen & bradykinine	(Leontovyč et al. 2018)
POP	*S. mansoni*	Member of serine peptidase family S9, cleavage of bradykinin and angiotensin I	(Fajtová et al. 2015)
CB1	*S. mansoni*	Degradation of TLR3 on macrophages, gut-associated peptidase, digestion of human blood cells	(Donnelly et al. 2010b) (Jílková et al. 2014)
GAPDH	*S. japonicum*	Induce short-lived antibody responses	(Wang et al. 2017)
FBA			
GST	*S. mansoni*	Stimulate anti-fecundity immunity	(Riveau et al. 1998)
HSP70	*S. mansoni*	Elicits an early and strong antibody response in baboons	(Kanamura et al. 2002)
Syntenin	*S. mansoni*	Vaccination of mice with recombinant syntenin confers partial protection against *S. mansoni* challenge infection and ameliorates parasite-induced liver pathology	(Figueiredo et al. 2014)
Sm29	*S. mansoni*	Activation of monocyte derived dendritic cells and lymphocytes in patients with leishmaniasis	(Lopes et al. 2019)

ATPase, adenosine triphosphatase; ATPDase, ATP diphosphohydrolase; CB1, cathepsin B-like endopeptidase 1; CE, cercarial elastase; FBA, fructose-bisphosphate aldolase; GAPDH, glyceraldehyde-3-phosphate dehydrogenase; GST, glutathione S-transferase; HSP70, heat shock protein 70 kDa; PEPCK, phosphoenolpyruvate carboxykinase; POP, prolyl oligopeptidase; Rab11, Rab-protein 11; Sj, *Schistosoma japonicum*; Sm, *Schistosoma mansoni*; Sm16, *Schistosoma mansoni* 16 kDa tegumental antigen; Sm22.6, *Schistosoma mansoni* 22.6 kDa tegumental antigen; Sm29, *Schistosoma mansoni* 29 kDa tegumental antigen; SNARE, Noluble N-ethylmaleimide-sensitive factor-attachment protein (SNAP) receptor; SPO-1 (Sm16), stage-specific protein-1; SP2, serine protease 2; TPI, triosephosphate isomerase; TSP-2, tetraspanin-2; VAMP2, vesicle-associated membrane protein 2; VIP36, vesicular integral membrane protein 36 kDa

## Adult schistosomes

An EV-based interaction between adult parasite and host was first described for *S. mansoni* in 1961 (Kuipers et al. 2018; Senft et al. 2006). They release cup-shaped exosome-like vesicles ranging from 50 to 130 nm size enriched in nucleic acid, proteins, cholesterol, and lipids (Samoil et al. 2018; Sotillo et al. 2016a). Exosome-like vesicles isolated from adult *S. japonicum* were found to have a typical spherical shape and a diverse population that varies in size of 30–100 nm (Wang et al. 2015). In this article we focus exclusively on molecules that have been identified as vesicles constitutes.

Lipids are critical components of exosomes and small extracellular vesicles. They build up a protective lipid bilayer that is directly exposed to the environment and represents the interacting surface with recipient host cells. The cargo of exosomes contains a variety of lipid types, including phosphatidylserine, sphingomyelin, cholesterol, and plasmalogen. Phospholipid lysophosphatidylcholine (LPC) increases the surface tension of the membrane and therefore influences exosome stability and function in vivo (Munder et al. 1979). LPC and prostaglandin (PG) D2, derived from *S. mansoni*, activate eosinophils via toll-like receptor 2 (TLR2), and prostaglandin D2 receptor 1 (DP1) fosters the release of TGF-β to support both fibrosis and tissue repair (Cao et al. 2016a, b; Nowacki et al. 2015a). The tegumental version of TLR2 promotes maturation of dendritic cells which in turn induce regulatory T cell development (van der Kleij et al. 2002).

Various proteome analyses have been conducted on E/S products of adult schistosomes. In contrast to mammals or bacteria, only a limited number of schistosomal E/S proteins contain signal peptides and are referred to as atypical or non-secretory, like HSPs, enolase, GAPDH, GST, 14-3-3 proteins, and a fatty acid binding protein (Liao et al. 2011). All these proteins have already been purified from schistosome vesicles and described as the most frequently secreted proteins in *S. japonicum* and *S. mansoni* (Samoil et al. 2018). Vesicle proteins of adult schistosomes include well-described exosomal markers designated in ExoCarta (Simpson et al. 2012), e.g., HSP70, energy-generating enzymes (e.g., enolase, pyruvate kinase, GAPDH, phosphoglycerate kinase 1), cytoskeletal proteins (actin, tubulin, fimbrin), tetraspanins (TSPs; e. g., TSP-1, TSP-4, TSP-18), and others (Knudsen et al. 2005; Deng et al. 2003). Proteomic analysis and classification by gene ontology (GO) annotation of vesicle contents of adult *S. mansoni* revealed a high incidence of proteins with catalytic and/or binding activity and proteins involved in metabolic and cellular processes. There is a great diversity of exosomal proteins but little is known about their function. *S. japonicum* exosomal enzymes induces classic activation of macrophages (M1), which produce pro-inflammatory mediators like TNF-α, CD 16/32, and inducible nitric oxide synthase (iNOS) (Wang et al. 2015).

Extracellular vesicle-enclosed microRNAs (miRNAs) of adult *S. mansoni* and *S. japonicum* were shown to play an essential role in modulating host immune responses (Samoil et al. 2018; Zhang et al. 2017; Cheng et al. 2013; Hoy et al. 2014) and act as important mediators of cell communication (Turchinovich et al. 2013; Hu et al. 2012). MicroRNAs (miRNAs) are short, highly conserved, non-coding RNA molecules that occur naturally in the genomes of plants and animals. The 17 to 27 long nucleotides target specific mRNAs and therefore regulate posttranscriptional mRNA expression. This results in translation suppression and gene silencing. At present, 79 mature miRNAs in *S. japonicum* and 225 mature miRNAs in *S. mansoni* have been documented in miRBase (Version 21) (Zhu et al. 2016). For *S. haematobium* 89 transcribed miRNAs were identified in total including 34 novel species specific, with no homologs in other schistosomes (Stroehlein et al. 2018). Research on the biological significance of exosomal microRNA is still in its beginnings. Computer-based searches for potential human target regions have revealed conserved seed regions in schistosomal miRNAs of *S. mansoni*. Sma-bantam and sma-miR-36-3P were found to be enriched in vesicles of adult *S. mansoni*. Sma-miR125b, one of the most abundant miRNAs found in *S. mansoni* EVs, has been shown to have more than 600 potential human targets. For sma-bantam 39 potential human targets have been identified (Samoil et al. 2018). Bantam is an invertebrate-specific miRNA that was previously detected in serum samples of helminth-infected hosts and was reported to be secreted by the parasite (Cheng et al. 2013; Hu et al. 2012; Britton et al. 2014). It has been shown that vesicular miR-125b and bantam-mirRNA from *S. japonicum* are taken up by macrophages and other peripheral host blood immune cells. Incorporated miR-125b and bantam increases macrophage proliferation and TNF-α production by regulating corresponding targets including Pros1, Fam212b, and Clmp and thus contributes to parasite survival (Liu et al. 2019). An in vitro study of Meningher et al. (2020) has shown that adult schistosomes secrete miRNA-harboring extracellular vesicles that are internalized by Th cells (Meningher et al. 2020). They also found schistosomal miRNAs in T helper cells isolated from Peyer’s patches and mesenteric lymph nodes of infected mice. As a target of MAP3K7, it has been shown that schistosomal miR-10 down-modulates NF-κB activity, a transcription factor for Th2 differentiation. Based on these results, the authors concluded that schistosomal miR-10 is involved in the downregulation of the Th2 response in the chronic course of the infection. Vesicle-packaged miRNAs were also tested as diagnostic tools and as a way to assess the severity of the disease. Meningher et al. (2017) could show that detection of two schistosomal miRNAs (bantam and miR-2c-3p) in sera of infected patients has comparable sensitivity (80%–86%) and the specificity (84%–94%) levels to commonly used serological tests (Meningher et al. 2017). In addition, the serum level of schistosomal miRNA has turned out to be a useful tool for the grading of hepatic fibrosis in Schistosomiasis (Cai et al. 2018). Four schistosomal miRNAs, miR-150-5p, let-7a-5p, let-7d-5p, and miR-146a-5p, have proven to be useful in this study to distinguish patients with mild versus severe fibrosis, with miR-150-5p as the most promising marker. In addition, all analyzed miRNAs have returned to normal levels in mice 6 months post-treatment.

Considering the localization of adult worms within the small mesenteric or pelvic veins, manipulation of vasodilatation and fibrinolysis represents an important adaption strategy of the parasite to the local environment. ATP-diphosphohydrolase 1 (ATPDase-1) is a membrane-associated protein that hydrolyzes extracellular prothrombotic ATP and ADP, thereby, inhibiting platelet aggregation and activation (Da’dara and Krautz-Peterson 2014; Kaczmarek et al. 1996; (Vasconcelos et al. 1996). Exosomal ATPDase-1 may represent an important mechanism of hemostatic control. Because of its plasminogen binding activity, the glycolytic enzyme enolase displays another exosomal protein with potential anti-clotting action (Gómez-Arreaza et al. 2014). The schistosomal antigen Sm22.6 is known to suppress the activity of host proteins like thrombin (Lin and He 2006), annexin (Madureira et al. 2011), and the calcium-dependent protease calpain. Vesicles containing parasite calpain alter calpain activity of the host and thus leading to impaired platelet aggregation (Kuchay and Chishti 2007).

Due to the production of eggs, female schistosomes ingest some 39.000 erythrocytes hourly (10 times more than male) (Figueiredo et al. 2015a, b), and digestion of blood constituents is largely extracellular (Skelly et al. 2014). The schistosomal esophagus is divided into an anterior and a posterior section. Each of these sections is surrounded by a dense assembly of glandular cell bodies displaying the origin of secretory vesicles. Once released into the intestinal lumen, they support digestion of blood (Li et al. 2014). Adult schistosomes habitually regurgitate their gut contents to expel the haemozoin. The vomitus contains a multitude of E/S proteins packed in exosome-like vesicles, e.g., hydrolytic enzymes required for the degradation of erythrocytes and host plasma constituents as well as carrier proteins promoting the uptake of lipids.

*S. mansoni* vesicles are also rich in proteases, including metallopeptidases, cysteine, and serine proteases that are believed to play important roles in exosome-mediated signaling (Shimoda and Khokha 2013). For example, novel serine proteases from family S1 trypsin-like named SmSP2 (Dvořák et al. 2016) and prolyl oligopeptidase from family S9 named SmPOP were shown to target bradykinin and angiotensin I (Fajtová et al. 2015). A homolog of leucine aminopeptidase (LAP), present in the adult worm gut, contributes to digestion of blood proteins (McCarthy et al. 2004). Some of these proteases and other tegumental proteins are promising vaccine candidates, including Sm-TSP-2 (Tran et al. 2006), Sm29 (Cardoso et al. 2006), and cytoplasmic dynein light chain (Rezende et al. 2011). Other proteases, e.g., cathepsin B-like peptidases, have immune regulatory activity. Macrophages of mice given a single injection of *S. mansoni* cathepsin B1 (SmCB1) differentiate into a Th2-associated M2 phenotype (Donnelly et al. 2010a). SmCB1 and other helminth cysteine peptidases act simultaneously as immunogens and adjuvants and are therefore interesting vaccine candidates (El Ridi et al. 2014). However, one of the major functions of this gut-associated peptidase is the digestion of human blood cells (Jílková et al. 2014).

Another very interesting vesicle-derived vaccine candidate is GAPDH of *S. japonicum*. It was identified as a major antigen inducing a short-term antibody response (Wang et al. 2013)*.* In different animal models, vaccination with GST of 28 kDa resulted in a significantly reduced fecundity of female worms (Liu et al. 2009; Riveau et al. 1998). Authors found that GST is the most abundant E/S protein of various parasites indicating highly conserved patterns of this protein. HSPs are also highly conserved and have been shown to be an immune stimulant such as SmHSP70 triggering an early humoral immune response and being a potential candidate for the use in immune diagnosis (Kanamura et al. 2002).

## Eggs

Parasite eggs release bioactive E/S products, which are known to be powerful modulators of the host immune response (Dvořák et al. 2016; Knuhr et al. 2018). Different proteomic analyses of *S. mansoni* egg secretions revealed protein numbers from six (Mathieson and Wilson 2010) to 188 (Cass et al. 2007). In *S. japonicum*, 258 (Liu et al. 2006) or rather 957 egg-related proteins (De Marco Verissimo et al. 2019) were found. Due to a lack of information, it remains elusive to what extent E/S products of eggs were secreted via vesicles. We found only one study that points to the release of miRNA-containing EVs by *S. japonicum* eggs, which can transfer their cargo miRNAs to recipient cells in vitro (Zhu et al. 2016), while others doubt EV-mediated secretions by schistosome eggs (Sotillo et al. 2016b).

## Summary

Vesicle contents of schistosomes facilitate life stage-specific requirements and biological processes like migration through the host body, feeding, or reproduction. However, the most important contribution of the vesicle-based communication is the promotion of parasite survival by modulating the immune response of the host (Han et al. 2009). By virtue of the strong immunological adaption of the host and the parasite, all attempts to develop a reliable anti-schistosomal vaccine have failed so far. Therefore, it is of crucial importance to understand and overcome the tightly regulated host-parasite “communication”. As described above, a certain number of vesicular schistosomal molecules have already been assigned a function and some of them, in particular proteins, are considered to be promising vaccine candidates or diagnostic markers.
